# Effect of focal muscle vibration on sEMG activity during repeated elbow movements in healthy adults

**DOI:** 10.1186/s12984-025-01816-4

**Published:** 2025-11-28

**Authors:** Carmen Cabezaolias, Rafael Raya, Cristina Sanchez, Rodrigo Rodriguez, Eloy Urendes

**Affiliations:** Departamento de Tecnologías de la Información, Escuela Politécnica Superior, Universidad San Pablo-CEU, CEU Universities, Urbanización Montepríncipe, 28660 Boadilla del Monte,, Spain

**Keywords:** Vibration therapy, Neuromuscular modulation, Surface electromyography, RMS, Reciprocal inhibition

## Abstract

**Background:**

Focal muscle vibration (FMV) is a non-invasive intervention that can modulate neuromuscular excitability, traditionally assessed with electrostimulation-evoked responses like F-waves or H-reflexes, which can be uncomfortable and impractical for many populations. Although the effects of FMV on targeted muscles have been well characterized, its impact on antagonist muscles, particularly during recovery after stimulation, remains poorly understood. Surface electromyography (sEMG) offers a non-invasive and clinically accessible method to assess these short-term changes and guide FMV-based interventions.

**Methods:**

30 healthy adults participated in a within-subjects crossover design with two sessions (control and FMV) separated by at least 24 h. Both sessions began with a baseline recording of triceps sEMG during repeated elbow flexion-extension movements to assess initial muscle activation. In the FMV session, focal vibration (120 Hz, 1.2 mm amplitude) was applied to the biceps brachii (agonist) for 5 min immediately after baseline, whereas in the control session, participants rested quietly for the same duration. In both sessions, triceps brachii (antagonist) activity was recorded using sEMG every 5 min over a 30-minute follow-up period, resulting in 7 trials. Root mean square (RMS) values were computed for each trial and expressed as post-baseline ratios. Intragroup effects were analyzed using linear mixed-effects models to account for repeated measures. Intergroup differences (FMV vs. control) were evaluated using paired t-tests or Wilcoxon signed-rank tests when normality was violated. All resulting p-values were adjusted using the Benjamini-Hochberg procedure to control the false discovery rate.

**Results:**

FMV induced a significant and sustained reduction in triceps RMS ratios, lasting up to 20 min post-intervention (*p* < 0.05). The control session showed no significant changes. Intergroup comparisons revealed lower RMS ratios in the FMV session at several follow-up time points (*p* < 0.05), which remained significant after the p-value adjustment.

**Conclusions:**

The findings support the hypothesis that FMV applied to the agonist muscle can transiently attenuate antagonist (triceps) muscle activity. sEMG proved to be a suitable and accessible method for detecting vibration-induced changes in muscle activation. This neuromodulatory effect may represent a promising tool for future rehabilitation or motor training strategies aimed at enhancing neuromuscular control and functional recovery.

## Background

Focal Muscle Vibration (FMV) is a noninvasive therapeutic technique that delivers mechanical vibratory stimuli directly to the belly of a targeted muscle. These rapid oscillations induce repetitive stretching of the muscle fibers. When the velocity exceeds the activation threshold of muscle spindle receptors, type Ia afferent fibers, highly sensitive to dynamic changes in muscle length, are predominantly stimulated [1]. This afferent input excites primary α-motoneurons in the spinal cord, leading to sustained involuntary contractions of the vibrated muscle, a response known as the Tonic Vibration Reflex (TVR) [2].

In addition to enhancing activity in the agonist muscle, Ia afferents engage spinal inhibitory interneurons that suppress activation in the antagonist muscle, a phenomenon known as reciprocal inhibition [3]. This mechanism reduces co-contraction, improves agonist efficiency, and enhances neuromuscular coordination [4]. Notably, this modulation may persist transiently after the vibration ends, reflecting short-term plastic changes in both spinal cord and supraspinal cord circuits [5]. However, despite the extensive evidence supporting these effects, the physiological mechanisms underlying vibration-induced neuromuscular responses remain insufficiently understood, as highlighted in recent reviews [6, 7].

Beyond these physiological mechanisms, FMV has been applied across diverse neurological conditions, demonstrating consistent therapeutic benefits [7]. In healthy individuals, FMV has been shown to enhance proprioceptive acuity and postural control through refined sensorimotor integration, contributing to improved coordination, joint stability, and movement precision [8–10]. Vibration-induced kinesthetic illusions of movement further demonstrate its capacity to engage the proprioceptive system and modulate body perception [11, 12]. Moreover, FMV has also been shown to produce a short-term analgesic effect and to reduce muscle soreness, likely mediated by the activation of large-diameter afferent fibers arising from the vibrated muscle [13, 14].

In individuals with stroke, it reduces spasticity and muscle co-contraction while improving motor coordination, voluntary control, and both upper and lower limb function, supporting its role in enhancing gait, dexterity, and overall motor recovery [15, 16]. In multiple sclerosis, the stimulation contributes to reduced lower-limb spasticity, improved gait stability, and increased endurance, supporting better overall mobility [17]. In patients with cerebral palsy, FMV improves postural control and balance by promoting reciprocal inhibition and enhancing muscle coordination, resulting in smoother and more efficient movement patterns [6]. In Parkinson’s disease, it has been reported to facilitate motor initiation and coordination, likely through modulation of proprioceptive feedback and cortical excitability [3].

The vibratory stimulus in FMV is primarily defined by two parameters: frequency and amplitude. Frequencies between 20 and 80 Hz can elicit the TVR at lower amplitudes but may have limited effects on reciprocal inhibition. In contrast, higher frequencies (above 80 Hz) enhance TVR strength, modify motor unit recruitment, and modulate antagonist muscle activity [18]. However, frequencies above 150 Hz may cause asynchronous muscle fiber discharge and discomfort, which limits their clinical applicability [19, 20].

Amplitude, defined as the maximum displacement of the vibrator from its resting (equilibrium) position, also plays a critical role. Amplitudes below 1 mm may be insufficient to activate muscle spindle receptors, while those exceeding 2 mm often lead to discomfort or overstimulation [21]. Therefore, FMV protocols typically use amplitudes in the 1–2 mm range [2].

High-frequency (80–150 Hz), low-amplitude (1–2 mm) protocols are the most employed in both research and clinical practice. In healthy individuals, FMV has been associated with improved balance (e.g., reduced center of pressure sway), increased muscle strength and endurance, and enhanced proprioceptive feedback during tasks involving postural control or maximal voluntary contraction [10, 22, 23]. Clinically, FMV has shown therapeutic effects in individuals with neurological conditions such as stroke, spinal cord injury, and multiple sclerosis, including reduced spasticity, measured via the Modified Ashworth Scale (MAS), improved joint range of motion, and better performance in functional tasks such as gait speed, the Box and Block Test, and the Timed Up and Go Test [24–27].

Nevertheless, most studies have focused on lower-limb and postural applications, while evidence regarding upper-limb motor recovery remains limited [7]. This gap highlights the need to explore FMV interventions aimed at enhancing manipulation and fine motor function in the upper extremities. Furthermore, as most studies assess FMV effects primarily through functional performance tests, future research should incorporate more objective and quantitative metrics to better elucidate the underlying neuromuscular mechanisms and validate the observed clinical outcomes [6].

To better understand FMV-induced adaptations, electrically evoked responses such as the H-reflex and F-wave have been widely used to assess spinal excitability and motoneuron responsiveness [3, 28]. Studies using these techniques suggest that FMV can potentially reduce antagonist activity via spinal inhibitory pathways [29]. These reflex-based techniques, typically measured using surface electromyography (sEMG), involve external electrical stimulation to elicit and record neural responses. While these methods provide valuable insight into spinal cord function, their reliance on electrical stimulation can cause discomfort, limit their applicability in certain clinical populations and makes them less suitable for studying voluntary movements or dynamic motor tasks.

In contrast, sEMG recorded without electrical stimulation offers a fully non-invasive, task-relevant and more comfortable alternative for evaluating muscle activity and neuromuscular function. Unlike H-reflex or F-wave techniques that infer spinal excitability from artificially evoked responses, sEMG directly captures the natural recruitment and modulation of motor units during functional movements. Importantly, these approaches are not directly comparable, as they reflect distinct aspects of neuromuscular function (reflex excitability versus voluntary activation), making their results complementary rather than equivalent. It can effectively capture changes in muscle activation patterns, offering meaningful data on FMV-induced neuromuscular adaptations.

To address this limitation, time-domain features of voluntary sEMG activity have gained attention as non-invasive alternatives. The root mean square (RMS) value, which represents the overall level of muscle activation over time, is widely used to quantify muscle activation and infer motor unit recruitment intensity [30, 31]. RMS has proven useful in assessing the effects of rehabilitation and stimulation protocols. In healthy participants, RMS reductions after interferential current therapy have been interpreted as evidence of increased neuromuscular efficiency due to reduced superficial muscle activation [32]. In clinical contexts, RMS has also helped quantify improvements following interventions such as functional electrical stimulation (FES), with studies reporting increased RMS values in paretic muscles, indicating enhanced voluntary recruitment [33]. While RMS provides a direct measure of activation amplitude, some studies complement it with spectral or time-frequency analyses to explore factors like motor-unit recruitment or conduction velocity. Recent research using high-density EMG during whole-body vibration interventions has further demonstrated its ability to characterize motor unit behavior and neuromuscular responses to vibratory stimuli [34].

Despite existing studies describing sEMG changes during or immediately after FMV, evidence on the temporal evolution of muscle activation in the minutes following stimulation remains limited. Notably, FMV applied to agonist muscle is associated with reductions in antagonist muscle activity after the vibration [35, 36].

This recovery window is of particular interest, as FMV is often applied to enhance the effectiveness of subsequent therapeutic interventions [14, 37]. Determining how these short-term neuromuscular effects evolve and decay over time is crucial, since the persistence of vibration-induced facilitation or inhibition directly influences motor performance and neuroplastic adaptation. Understanding this temporal profile allows clinicians to schedule rehabilitation sessions when the neuromodulatory effects are still active and to avoid potential overlap or desensitization from repeated stimulation.

Previous studies have investigated the duration of vibration-induced effects using mainly functional or subjective outcome measures, which, although clinically relevant, are less sensitive and precise than physiological, quantitative assessments [6]. However, the optimal time and underlying mechanisms remain unclear. For instance, Noma et al. [38] demonstrated that five minutes application of high-frequency, low-amplitude vibration applied to the agonist muscle could induce changes in its neuromuscular excitability lasting up to 30 min, as shown by F-wave responses. These findings offer valuable insight into how vibration may modulate the excitability of agonist motoneurons. Nevertheless, this study once again relies on electrically evoked responses, an approach whose limitations have been previously noted.

Accordingly, the present study aimed to characterize short-term neuromuscular modulation induced by FMV, moving beyond reflex-based paradigms by employing voluntary, non-stimulated sEMG recordings. Specifically, it examined the evolution of antagonist (triceps brachii) activity following FMV applied to the agonist (biceps brachii). In addition, this study sought to obtain an objective metric through sEMG analysis, since most previous research has relied primarily on functional performance outcomes rather than direct electrophysiological measures [6]. Given the goal of assessing global modulation of muscle activity over time, RMS time-domain analysis, rather than frequency-domain approaches, was selected as a robust and clinically interpretable measure, since amplitude-based estimators such as RMS provide a physiologically meaningful representation of motor-unit recruitment during voluntary contractions [39, 40].

A within-subject, repeated-measures design was employed, in which participants completed both control and FMV sessions on separate days. sEMG was used to quantify triceps brachii activity during repeated elbow flexion-extension in the sagittal plane, and RMS values were normalized to baseline (pre) measurements recorded at the beginning of each session to assess post-baseline changes. Within-condition (pre vs. each post-trial within each group) and between-condition (control vs. FMV) comparisons were performed across seven time points over a 30-minute follow-up period. Overall, the objective was to determine whether FMV induces a measurable, transient reduction in antagonist muscle activation, assessed through non-invasive and clinically applicable protocol.

## Methods

### Participants

30 healthy adults (mean age 32.8 ± 8.9 years; 18 males and 12 females) were voluntarily recruited for the present experiment. A sample size of 30 participants was selected based on prior studies with similar within-subject designs and was deemed sufficient to detect medium-to-large effects while maintaining feasibility [9, 22, 23, 29]. Inclusion criteria included ages between 18 and 50 years, to avoid age-related muscle fiber loss [41], and no history of neurological disorders or injuries. All participants received written information about the study and provided informed consent prior to testing. This single-center study received ethical approval from the Research Ethics Committee of CEU San Pablo University, Madrid, Spain (reference number: 1029-25 137).

### Instrumentation

**Fig. 1:**
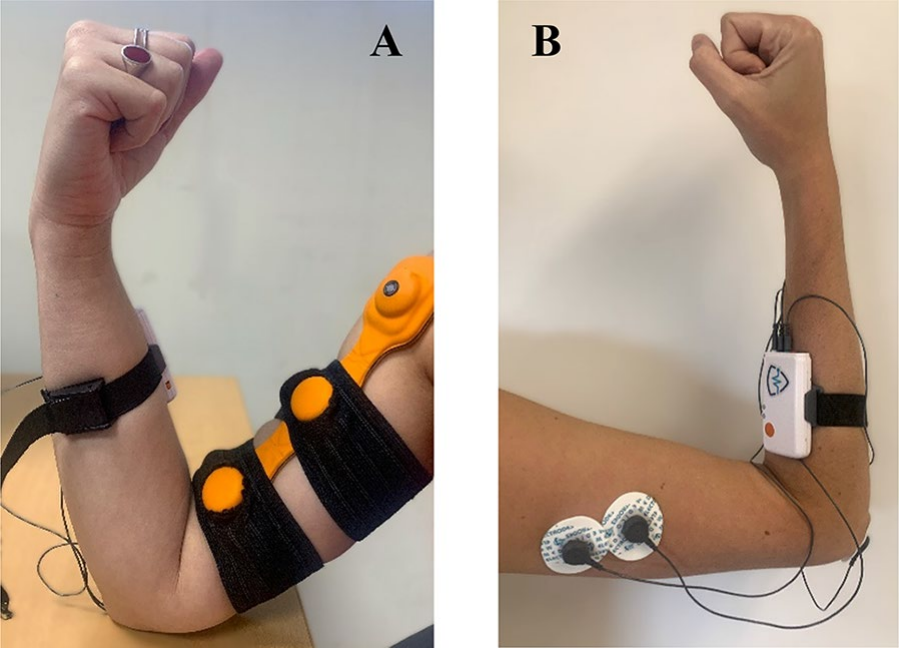
FMV and surface electrode placement for the experimental setup. **A** Placement of FMV device (Myvolt™ Arm) over the biceps brachii. **B** sEMG electrode placement for the triceps branchii following SENIAM guidelines

The vibratory device used in this study was the Myovolt™ Arm (Myovolt Ltd., New Zealand), which consists of two integrated actuators embedded in a soft, flexible material. This wearable device supports three vibration modes: sinusoidal, pulsed, and continuous. The continuous mode was selected, as it provides high-frequency (120 Hz) and low-amplitude (1.2 mm) mechanical stimulation directly to the muscle, in line with the parameter range previously identified as effective or eliciting reciprocal inhibition effects in antagonist muscles [42]. The frequency of 120 Hz was specifically chosen to elicit a strong TVR and facilitate reciprocal inhibition while remaining comfortable for participants [18]. The device was secured over the skin surface of the biceps brachii muscle using an adjustable elastic strap with two openings to accommodate the actuators. This configuration ensured stable skin contact without compressing the muscle, allowing consistent vibration delivery throughout the stimulation period, as shown in Fig. 1A.

The mDurance™ system (mDurance Solutions, Spain) is a wireless dual-channel device designed for non-invasive assessment of muscle activity [43]. Only one channel was used to collect sEMG data from the triceps brachii. The system includes an integrated inertial measurement unit (IMU) for tracking range of motion (ROM) within the movement in the sagittal plane. Data were visualized in real time via the mDurance™ mobile app, which also features a built-in metronome to standardize movement rhythm during dynamic tasks. Additionally, its cloud-based server interface enabled remote monitoring and facilitated the download of raw, filtered sEMG and ROM data.

For each participant, three pre-gelled surface electrodes were placed according to SENIAM guidelines: two on the long head of the triceps brachii and one reference electrode on the bony surface of the elbow, as shown in Fig. 1B [44]. This electrode configuration ensured reliable signal acquisition from antagonist muscle during the motor task. Electrodes were connected to the mDurance™ control unit, which was secured to the forearm using an elastic strap. Continuous data acquisition and monitoring were conducted through the accompanying mobile application.

### Procedure

Each participant completed 2 experimental sessions: a control session and an FMV session, conducted on separate days with a minimum interval of 24 h to avoid carryover effects. Both sessions began with electrode placement following standardized procedures. Electrodes remained fixed throughout the same session to ensure consistent signal acquisition.

An initial baseline measurement (pre) was taken in each session. Participants performed repeated elbow flexion-extension movements with their dominant arm for one minute at a controlled pace of 60 beats per minute (bpm), guided by a metronome. This task was selected due to its simplicity as a single-joint motion and its suitability for evaluating motor control in healthy adults, being minimally affected by age-related variability [45]. The velocity of 60 bpm was chosen because recent studies have demonstrated that this cadence allows for stable joint kinematics and consistent assessment of motor control while minimizing variability in joint configuration despite changes in muscle activation [46, 47]. All participants were seated upright with back support, knees flexed at 90°, and the tested arm free of external load or resistance.

In the control session, following the initial baseline measurement, participants rested for five minutes, mimicking the FMV time period, before completing seven additional trials (post) of the same flexion-extension task. Each trial lasted one minute, with a four-minute rest interval between sets, a duration recommended to minimize fatigue during repeated dynamic task [48]. In the FMV session, after the baseline measurement, FMV was applied to the biceps brachii for five minutes. During this period, the participant’s arm was supported on a desk in maximum flexion to ensure stable contact and comfort, as previously shown in Fig. 1A. This position allowed consistent contact and uniform transmission of the mechanical stimulus. Immediately after FMV, the same sequence of seven flexion-extension trials was repeated under identical timing and posture conditions as in the control session.

**Fig. 2:**
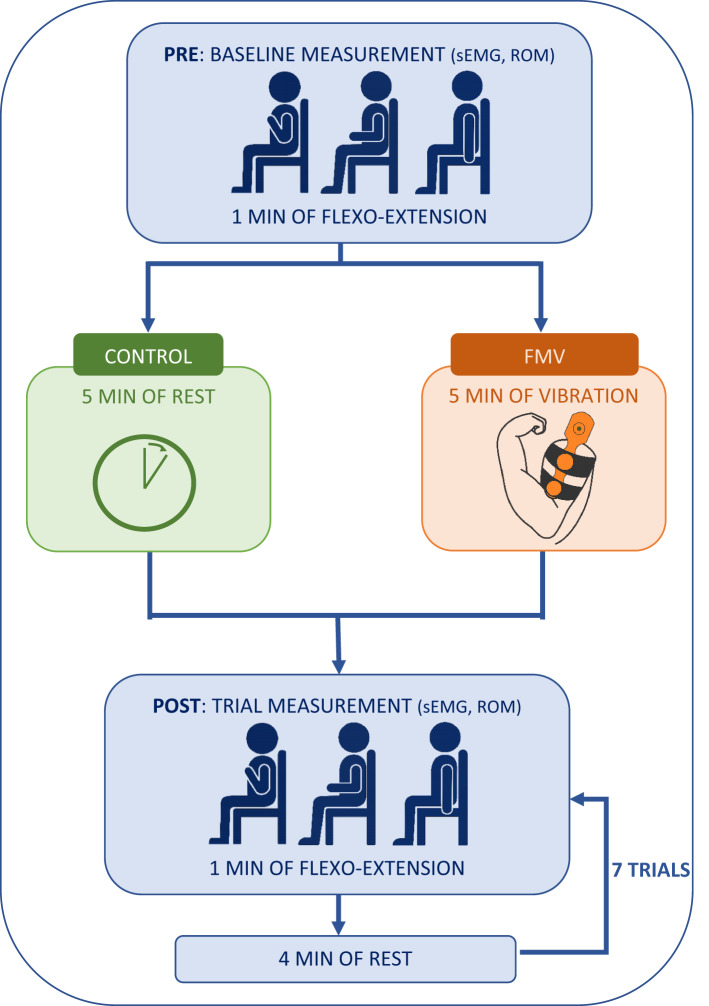
Experimental protocol overview. Participants completed two sessions (control and FMV), each starting with a baseline (pre) measurement during repeated elbow flexion-extension. This was followed by either 5 minutes of rest (control in green) or focal vibration (FMV in orange). Afterward, participants performed seven 1-minute flexion-extension trials, each separated by 4-minute rest periods. ROM and triceps brachii sEMG were recorded throughout. Blue indicates procedures common to both sessions

sEMG and ROM data were recorded from the triceps brachii during all movement repetitions. This design allowed the analysis of muscular activity both in the absence of stimulation (control session) and in response to the FMV intervention (FMV session). Neuromuscular activity was monitored during the 30 min follow-up period, consistent with the typical therapeutic window used in manual interventions for individuals with neurological conditions [49]. The full experimental protocol is illustrated in Fig. 2.

### Signal processing

Muscle activity was recorded using sEMG throughout all elbow flexion-extension trials. Raw sEMG signals were sampled at 1024 Hz and processed using a fourth-order Butterworth bandpass filter with a passband of 20–450 Hz to attenuate movement artifacts and high-frequency noise. The filtered signals were then rectified and smoothed using a 0.25-second RMS sliding window, yielding an envelope signal with an effective sampling rate of 4 Hz, in accordance with standard procedures for RMS-based sEMG analysis [43]. Maximal voluntary contraction (MVC) normalization was omitted to ensure applicability in clinical populations (e.g., individuals with cerebral palsy), where reliable MVCs are often unfeasible due to spasticity, hypotonia, or limited voluntary control [50].

All signal processing was performed using Python 3 (Jupyter Notebook). To account for baseline noise and ensure accurate detection of muscle activation, a baseline correction procedure was applied. This involved identifying the minimum RMS value prior to movement onset and defining a 60-point window around this minimum. Within this window, the mean and standard deviation were computed, and a muscle-specific activation threshold was defined as the mean plus three standard deviations. This threshold was subtracted from the entire signal, and only values above it were retained for analysis. This method, adapted from Sarcher et al. [50], enhances sensitivity to true muscle activity while minimizing the contribution of resting noise.

For each 1-minute recording, a subset of 10 consecutive flexion-extension cycles was extracted from the central portion of the movement period, based on the ROM signal captured by the mDurance™ system. This selection was made to avoid transitional effects at the beginning and end of the recording.

For each participant and session, the total RMS value across these 10 was computed. To quantify changes in antagonist activation, triceps RMS values at each follow-up time point (post) were normalized to initial baseline (pre) by computing post/pre ratio. These ratios were then used in both intra-group (time-based) and inter-group (condition-based) statistical comparisons. This approach was appropriate given the study’s focus on relative, time-dependent changes in activation rather than absolute amplitude values, making additional normalization unnecessary.

### Statistical analysis

All statistical analyses were conducted using Python 3 (Jupyter Notebook), with statistical significance set at *p* < 0.05. Two complementary statistical approaches were used to assess the effects of FMV on antagonist muscle activity: within-condition comparisons over time, and between-condition comparisons at each time point.

Within-condition (intragroup) analyses were performed using Linear Mixed Models (LMMs), which are well-suited for repeated-measures data as they account for correlations within subjects. Random effects were included to model individual variability and ensure robustness in the presence of missing or unbalanced data. Post-hoc comparisons were conducted between the baseline (pre) and each follow-up time point (post) within each session (control and FMV). To address multiple comparisons, p-values were adjusted using the Benjamini–Hochberg procedure to control the false discovery rate (FDR).

Between-condition (intergroup) comparisons between FMV and control sessions were made at each follow-up time point using paired samples t-tests. Normality of the paired differences was first assessed using the Shapiro–Wilk test. Depending on the result, either a paired t-test (for normally distributed differences) or a Wilcoxon signed-rank test (for non-normal distributions) was applied. Multiple comparisons were again corrected using the Benjamini–Hochberg procedure to maintain FDR control across tests.

## Results

Intragroup comparisons were performed using LMMs to assess differences in triceps brachii RMS ratios at each follow-up time point (post) relative to baseline (pre), separately for the control and FMV sessions. P-values were adjusted using the Benjamini-Hochberg procedure to control the false discovery rate. As shown in Table 1, no statistically significant differences were found in the control session across any follow-up time point (adj. *p* > 0.05 for all comparisons). In contrast, the FMV session exhibited significant reductions in RMS ratios at Post (adj. *p* = 0.002), Post 5 (adj. *p* = 0.002), Post 10 (adj. *p* = 0.004), Post 15 (adj. *p* = 0.002), and Post 20 (adj. *p* = 0.002). No significant differences were detected at Post 25 or Post 30 (adj. *p* = 0.431 and adj. *p* = 0.131, respectively).

**Table 1:** Intragroup comparison of triceps brachii RMS ratio across time points

Follow-up point (min)	Session	RMS Ratio (Mean ± SD)	Adj. *p*-values
Post	Control	1.08 ± 0.37	0.581
	FMV	0.84 ± 0.32	**0.002**
Post 5	Control	1.10 ± 0.39	0.581
	FMV	0.80 ± 0.29	**0.002**
Post 10	Control	1.06 ± 0.35	0.936
	FMV	0.86 ± 0.37	**0.004**
Post 15	Control	1.13 ± 0.45	0.581
	FMV	0.84 ± 0.33	**0.002**
Post 20	Control	1.15 ± 0.55	0.581
	FMV	0.85 ± 0.35	**0.002**
Post 25	Control	1.15 ± 0.61	0.581
	FMV	0.97 ± 0.46	0.431
Post 30	Control	1.04 ± 0.37	0.939
	FMV	0.93 ± 0.42	0.131

“Follow-up point (min)” indicates the time at which each trial measurement was taken. Mean ± SD values represent the RMS ratios normalized to baseline (Pre) within-condition in each session (Control and FMV). Adjusted p-values, derived from Benjamini-Hochberg adjustment from the LMM, indicate differences from baseline. Significant values (*p* < 0.05) are in bold

For intergroup comparisons between the control and FMV sessions at each follow-up time point, paired-samples t-tests were performed (degrees of freedom: *n – 1 = 29*). The normality of paired differences was assessed using the Shapiro–Wilk test, and all comparisons satisfied the assumption of normality; therefore, paired t-tests were used for all time points. P-values were adjusted using the Benjamini-Hochberg procedure to control the false discovery rate. As presented in Table 2, statistically significant differences between conditions were found at Post (adj. *p* = 0.017), Post 5 (adj. *p* = 0.013), Post 10 (adj. *p* = 0.033), Post 15 (adj. *p* = 0.013), and Post 20 (adj. *p* = 0.017). No significant differences were detected at Post 25 or Post 30 (adj. *p* > 0.4).

**Table 2:** Statistical comparison of triceps brachii RMS ratio between control and intervention sessions

Follow-up point (min)	Adj. *p*-value
Post	**0.017**
Post 5	**0.013**
Post 10	**0.033**
Post 15	**0.013**
Post 20	**0.017**
Post 25	0.426
Post 30	0.426

Statistical comparison of triceps brachii RMS ratios between control and FMV sessions at each follow-up point. Paired-samples t-tests were conducted after confirming the normality of paired differences using the Shapiro–Wilk test. P-values were adjusted using the Benjamini–Hochberg procedure to control the false discovery rate. statistically significant values (*p* < 0.05) are shown in bold

**Fig. 3:**
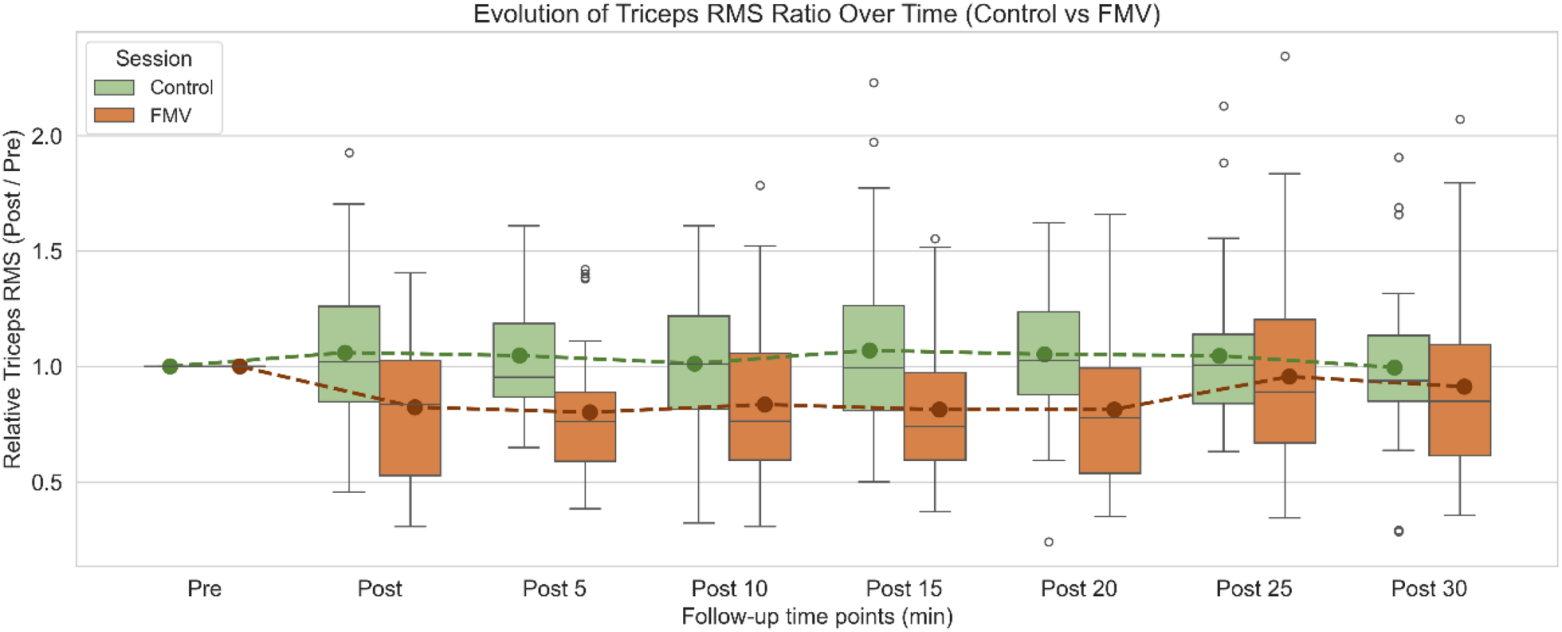
Evolution of Triceps RMS Ratio Over Time (Control vs FMV) Boxplots of triceps brachii RMS ratio (Post/Pre) across time for both sessions. Green represents the control session and orange the FMV session. Each boxplot shows the distribution of individual responses at each time point, with solid-colored points indicating group means and dashed lines connecting them over time. Data illustrates the temporal progression of triceps muscle activation normalized to baseline across the 30-minute follow-up period

Figure 3 presents boxplots of the normalized triceps RMS ratio (Post/Pre) for each time point in both the control (green) and FMV (orange) conditions. Solid points represent group means, and dashed lines connect them across time. As shown, the FMV session exhibited lower RMS ratios than control from Post to Post 20, reflecting the results reported in Tables 1 and 2. After Post 20, values in both groups approached baseline levels.

## Discussion

This study examined the short-term neuromuscular effects of FMV applied to the biceps brachii and its modulatory influence on the antagonist muscle, the triceps brachii, during a dynamic elbow flexion-extension task. Using sEMG and analyzing RMS ratios, we aimed to quantify antagonist muscle activation over time and across conditions within a fully non-invasive and clinically applicable framework.

The main finding was a significant reduction in triceps RMS ratios in the FMV session compared to both the pre-intervention baseline and the control session. This inhibition was evident immediately following the FMV application and persisted for up to 20 min. These results are consistent with the hypothesis that FMV induces spinal reciprocal inhibition, whereby Ia afferent input from the vibrated agonist muscle suppresses antagonist motoneuron activity via interneuron circuits. The transient RMS reduction likely reflects short-term changes in motoneuron excitability and motor-unit recruitment efficiency, consistent with previous FMV studies showing that vibration applied to the agonist muscle decreases spinal excitability and reduces α-motoneuron responsiveness [38].

Building upon these results, the present study introduces a quantitative approach based on sEMG-derived RMS ratios obtained during voluntary movement, offering a movement-based yet objective alternative to existing evaluation methods. Whereas electrically evoked responses such as the H-reflex or F-wave provide precise but invasive information about spinal excitability, and functional performance tests capture overall outcomes but lack objectivity and physiological specificity, the proposed metric bridges these two perspectives [6, 28]. By referencing post-intervention activity to the pre-intervention baseline, this measure enables the detection of vibration-induced modulation within a natural motor context, yielding a pain-free, objective, and clinically applicable tool to complement both traditional neurophysiological and functional assessments.

Our findings parallel those of Noma et al. [38], who demonstrated prolonged changes in the agonist muscle following five minutes of FMV. However, their study focused on the TVR reflex and employed electrical stimulation for assessment. In contrast, the present work extends those insights by demonstrating that inhibitory effects can be captured in the antagonist muscle using sEMG-derived RMS ratios during movement, providing a more functional and non-invasive approach [30].

The observed suppression of triceps activation suggests the existence of a temporary window of altered excitability lasting up to 20 min following FMV. This period may be clinically relevant, as FMV is increasingly employed as a preparatory or priming technique to enhance the effectiveness of subsequent motor training or rehabilitation interventions [14]. Notably, triceps RMS values began returning toward baseline at 25 and 30 min post-FMV intervention, indicating that the neuromodulatory effects of FMV are transient and time dependent. These findings underscore the importance of timing therapeutic interventions to align with the period of enhanced neuromuscular responsiveness, particularly given previous evidence suggesting that rehabilitation outcomes may be improved when applied shortly after vibration.

The control session confirmed that the changes in muscle activation were not due to repeated movement, fatigue, or procedural artifacts. In the absence of FMV, triceps RMS ratios remained statistically stable across all time points, underscoring the specificity of the vibration-induced effects. Furthermore, consistent electrode placement according to SENIAM guidelines, fixed positioning throughout the same session, and systematic preprocessing (including baseline correction and normalization) helped minimize variability and enhance signal reliability.

Despite its strengths, this study has several limitations that warrant consideration. RMS analysis offered a reliable and appropriate measure of muscle activation for the study’s aims. Although RMS amplitude can be influenced by non-neural factors such as electrode positioning, subcutaneous tissue thickness, or motor-unit synchrony, these effects were minimized in the present study by using within-subject normalization (post/pre ratios). This approach ensured that any potential variability due to recording conditions was consistent across trials, allowing changes to be interpreted primarily as neuromuscular adaptations rather than technical artifacts. While sufficient for assessing vibration-induced modulation, future studies may consider additional metrics such as co-activation indices or performance-based measures, to broaden the scope of neuromuscular assessment. Moreover, the study examined only a single FMV protocol; additional research is needed to assess the effects of varying frequencies, amplitudes, and durations. Finally, as the findings are based on healthy participants, they should be interpreted within this context, and validation in clinical populations is required before broader generalization.

Future studies should extend this work to clinical populations, particularly individuals with spasticity, to determine whether the inhibitory effects of FMV observed in healthy adults translate into clinically meaningful reductions in muscle tone. Investigating the integration of FMV with task-specific rehabilitation may further clarify its functional benefits and long-term impact. Although no participants in the present study reported discomfort, individuals with neurological conditions may present altered sensory or pain sensitivity. Therefore, future applications should carefully adjust vibration parameters such as frequency and amplitude to ensure both therapeutic effectiveness and patient comfort. Together, these directions support the use of FMV as both a research tool and a feasible neuromodulatory technique for real-world rehabilitation programs.

## Conclusions

This study demonstrates that five minutes of high-frequency, low-amplitude FMV applied to the biceps brachii produces a measurable and transient reduction in antagonist (triceps) muscle activity during dynamic movement. This inhibitory effect is specific to the intervention and lasts up to 20 min post-application. By leveraging non-invasive sEMG and RMS analysis, the study provides a practical method to monitor neuromuscular responses to FMV. These findings support the role of FMV as a viable priming tool in rehabilitation settings and underscore the importance of aligning therapy timing with the window of neuromuscular modulation induced by vibration. Future studies should determine whether these inhibitory effects observed in healthy adults extend to clinical populations and contribute to meaningful reductions in spasticity.

## Data Availability

The datasets generated and analyzed during the current study are available in the Zenodo repository, [https://doi.org/10.5281/zenodo.16030243] .The software was developed in Jupyter Notebook 7 (Python 3). The code supporting the conclusions of this article are available from the corresponding author upon reasonable request.

## Abbreviations

FMV: Focal Muscle Vibration
sEMG: Surface Electromyography
TVR: Tonic Vibration Reflex
RMS: Root Mean Square
ROM: Range of Motion
BPM: Beats per Minute
MVC: Maximal voluntary contraction
LMMs: Linear Mixed Models
FDR: False Discovery Rate
